# Safety, immunogenicity, and optimal dosing of VLPCOV-02, a SARS-CoV-2 saRNA vaccine with modified 5-methylcytosine base

**DOI:** 10.1016/j.isci.2026.114766

**Published:** 2026-01-21

**Authors:** Masayuki Aboshi, Daisuke Kawakami, Kaoru Kono, Ayae Nishiyama, Takuto Nogimori, Yuko Sunada, Kenta Matsuda, Takashi Sekida, Shigeru Suga, Jonathan F. Smith, Nobuaki Sato, Takuya Yamamoto, Wataru Akahata

**Affiliations:** 1 VLP Therapeutics Japan, Inc., 1-2-9 Nishi-Shinbashi, Minato-ku, Tokyo 105-0003, Japan; 2 Laboratory of Precision Immunology, Center for Intractable Diseases and ImmunoGenomics, National Institutes of Biomedical Innovation, Health, and Nutrition, Osaka 567-0085, Japan; 3 VLP Therapeutics, Inc., Gaithersburg, MD 20878, USA; 4 National Hospital Organization, Mie National Hospital, Tsu, Mie 514-0125, Japan; 5 Laboratory of Aging and Immune Regulation, Graduate School of Pharmaceutical Sciences, The University of Osaka, Osaka 565-0871, Japan; 6 Department of Virology and Immunology, Graduate School of Medicine, The University of Osaka, Osaka 565-0871, Japan

**Keywords:** Immunology

## Abstract

Variant-adapted vaccines are becoming increasingly important for continued COVID-19 prevention. Part 1 of the phase 1/2 study with VLPCOV-02, a lipid nanoparticle-encapsulated, self-amplifying RNA (saRNA) vaccine with a modified 5-methylcytosine (5 mC) base, demonstrated lower reactogenicity and incidence of adverse events, and induction of antibody responses. We report results of part 2 with an expanded number of participants (*N* = 323 [3 μg VLPCOV-02: 53 non-elderly and 54 elderly; 7.5 μg VLPCOV-02: 55 non-elderly and 55 elderly; 30 μg Comirnaty ready to use: 52 non-elderly and 54 elderly]) to determine the optimal dose level. VLPCOV-02 induced robust immunoglobulin G titers against receptor-binding domain and neutralizing antibody titers against all variants of SARS-CoV-2 tested, and induced CD4^+^ and CD8^+^ T cell responses. These results indicate that the incorporation of a modified 5 mC base improves the safety profile of the saRNA vaccine without compromising immunogenicity, supporting further development of this platform as a booster vaccine.

## Highlights

• 5 mC-incorporated VLPCOV-02 improved safety without compromising immunogenicity
• VLPCOV-02 induced strong, durable neutralizing antibodies up to 52 weeks
• An optimal dose of 3 μg VLPCOV-02 achieved immunity comparable to 30 μg BNT162b2
• VLPCOV-02 induced robust CD4^+^ Th1 and CD8^+^ T cell responses across age groups

## Graphical abstract

**Figure:**
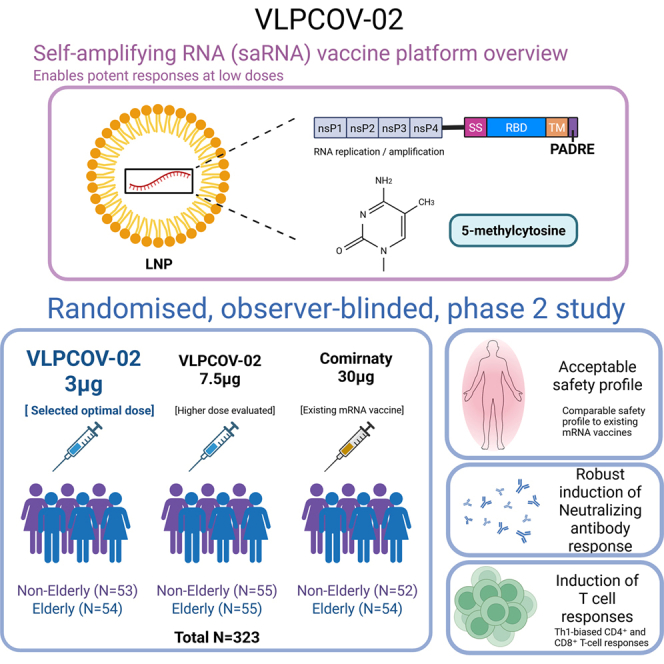

## Introduction

Coronavirus disease 2019 (COVID-19), caused by severe acute respiratory syndrome coronavirus 2 (SARS-CoV-2), has resulted in approximately 777 million cases and over 7 million deaths worldwide since the World Health Organization (WHO) declared it a global pandemic nearly 5 years ago.^1^ The pandemic triggered the rapid development and rollout of SARS-CoV-2 vaccines, resulting in 12 COVID-19 vaccines with four distinct vaccine platforms (protein subunit, RNA-based, non-replicating viral vector, and inactivated virus) being granted Emergency Use Listing by the WHO.^2^ SARS-CoV-2 vaccines have demonstrated an ability to effectively elicit humoral and cellular immune responses, decreasing severe infections, hospitalizations, and mortality, with an estimated 19.8 million lives saved during the first year of COVID-19 vaccine rollouts alone.^3^^,^^4^ While no longer considered a global public health emergency, vaccination against SARS-CoV-2 will be critical for continued disease prevention in the future, whereby it will be important to deploy variant-adapted vaccines in response to the emergence of new variants.

A lipid nanoparticle (LNP)-encapsulated, self-amplifying RNA (saRNA) vaccine platform was developed with a modifiable antigenic domain, allowing for expedited vaccine development to address emerging variants of concern.^5^^,^^6^^,^^7^ Due to the ability to amplify itself once delivered to cells and to present antigens for prolonged periods, saRNA vaccines have the capacity to induce durable immunity at significantly lower doses compared to non-amplifying messenger RNA (mRNA) vaccines.^8^^,^^9^ Furthermore, the lower dosage required to elicit immune responses helps to mitigate against the potential for reactogenic responses that are associated with the inflammatory properties of LNPs, which can trigger symptoms through cytokines, such as interleukin (IL)-1β and IL-6.^10^ There are also benefits associated with lower dosages, such as reduced cost of vaccine and increased scalability, which may make the vaccine more accessible in resource-limited countries.

VLPCOV-01 is an LNP-encapsulated saRNA COVID-19 vaccine that expresses a membrane-anchored receptor-binding domain (RBD) derived from the SARS-CoV-2 (wild type) spike protein.^5^^,^^6^ In a phase 1 booster study of VLPCOV-01, strong immune responses were observed following administration to healthy adults who had already received primary vaccination with Comirnaty (BNT162b2). Importantly, the responses induced by VLPCOV-01 were achieved at lower than one-tenth of the Comirnaty dose, and results suggested a potentially longer duration of response.^5^

Following the VLPCOV-01 phase 1 trial, the structure of VLPCOV-01 was revised by incorporating the pan human leukocyte antigen – DR isotype-binding epitope sequence at the 3′ end of the transmembrane sequence to augment the vaccine response, and a modified 5-methylcytosine (5 mC) base to reduce reactogenicity.^7^ A preclinical *in vivo* and *in vitro* study on the 5 mC-incorporating saRNA platform showed prolonged and robust antigen expression with reduced type-I interferon (IFN-I) induction in peripheral blood mononuclear cells (PBMCs) *in vitro* and robust immunoglobulin G (IgG) responses against SARS-CoV-2 RBD in immunized mice, supporting the potential clinical use of this platform with reduced adverse effects.^6^ Additionally, the RBD sequence was updated to be derived from the SARS-CoV-2 Gamma variant.^7^ According to a modeling study, it has been suggested that variant-modified vaccines may offer additional protection when compared with ancestral booster vaccines, even if they are not specifically matched to the currently circulating variants.^11^

The modified vaccine, VLPCOV-02, was evaluated in a phase 1 dose-escalation booster vaccination study, and elicited a robust increase in serum anti-RBD IgG titers among healthy Japanese adults who received prior immunization with an approved SARS-CoV-2 vaccine, demonstrating that the incorporation of 5 mC did not impair immunogenicity. Furthermore, VLPCOV-02 had a favorable safety profile and appeared to be less reactogenic than the non-base-modified VLPCOV-01, as suggested by comparing adverse events observed at equivalent vaccine doses in their respective phase 1 studies, where VLPCOV-02 showed fewer reactogenic effects than those previously reported for VLPCOV-01.^5^^,^^7^

Here, we present part 2 of the phase 1/2 study, where the aim was to determine the optimal dose of VLPCOV-02 and compare its safety and immunogenicity profile to the commercially available mRNA vaccine Comirnaty (Bivalent: Wild type/Omicron BA.4-5).

## Results

### Participants and baseline characteristics

**Figure 1:**
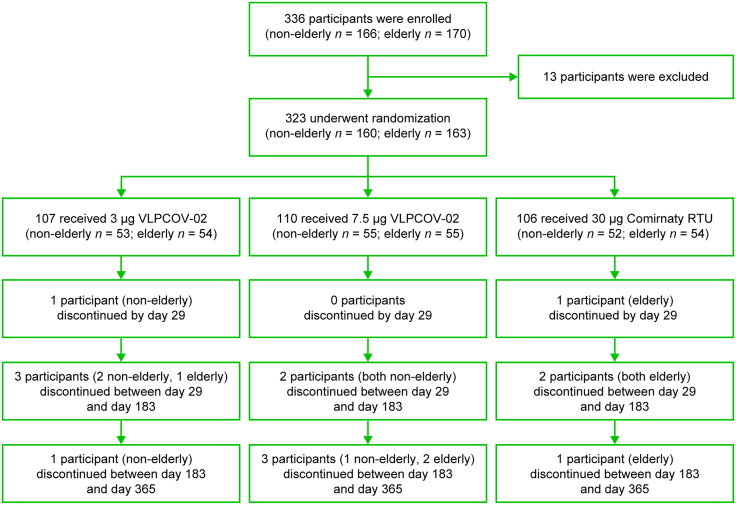
Participant randomization Study schema to show participant enrollment and randomization. Participants underwent randomization between June 25, 2023, and July 21, 2023. At 4 weeks post-study drug administration, two participants discontinued the study. By 52 weeks, an additional 12 participants discontinued from the study. All other participants completed the study through 52 weeks.

A total of 323 participants underwent randomization between June 25, 2023, and July 21, 2023. Of the 323 participants who received booster injections, 107 (non-elderly, 53; elderly, 54) participants received 3 μg VLPCOV-02, 110 (non-elderly, 55; elderly, 55) participants received 7.5 μg, and 106 (non-elderly, 52; elderly, 54) participants received 30 μg Comirnaty ready to use (RTU). At 4 weeks, two participants discontinued the study: one participant in the 3 μg VLPCOV-02 cohort for reasons unrelated to the study drug (inability to attend the study site due to relocation), and one participant in the Comirnaty RTU cohort due to the participant withdrawing consent. Between 4 weeks (day 29) and 52 weeks (day 365) after study drug administration, a total of 12 participants were discontinued from the study. The reasons for discontinuation included withdrawal of consent (three participants in the VLPCOV-02 group), the principal investigator or sub-investigator determined that adherence to the study protocol was no longer feasible (one participant in the VLPCOV-02 group), the participant was unable to attend visits (two participants in the VLPCOV-02 group), or if the participant received or requested the administration of an already approved SARS-CoV-2 vaccine following study drug administration (three participants in the VLPCOV-02 group and three participants in the Comirnaty RTU group). All other participants completed the study through 52 weeks (Figure 1). The demographic characteristics of the participants at enrollment (Per-Protocol-Set) are shown in Table 1. The number of participants in each analysis set (Full-Analysis-Set, Per-Protocol-Set, and Safety-Evaluable Set) and the reasons for exclusion are included in Table 2.

**Table 1:** Baseline demographic and characteristics for the per-protocol set

	Non-elderly			Elderly			Overall		
	VLPCOV-02		Comirnaty RTU	VLPCOV-02		Comirnaty RTU	VLPCOV-02		Comirnaty RTU
	3 μg	7.5 μg	30 μg	3 μg	7.5 μg	30 μg	3 μg	7.5 μg	30 μg
	*N* = 48	*N* = 50	*N* = 50	*N* = 51	*N* = 53	*N* = 49	*N* = 99	*N* = 103	*N* = 99
**Age (years)**									

Mean (SD)	52.9 (8.8)	51.9 (7.9)	50.4 (8.2)	69.1 (3.6)	69.8 (3.5)	71.0 (3.7)	61.3 (10.5)	61.1 (10.8)	60.6 (12.1)
Median (range)	55 (24–64)	53 (24–64)	50 (26–64)	69 (65–79)	69 (65–78)	71 (65–80)	65 (24–79)	65 (24–78)	64 (26–80)

**Gender, n (%)**									

Male	26 (54.2)	28 (56.0)	25 (50.0)	29 (56.9)	33 (62.3)	26 (53.1)	55 (55.6)	61 (59.2)	51 (51.5)
Female	22 (45.8)	22 (44.0)	25 (50.0)	22 (43.1)	20 (37.7)	23 (46.9)	44 (44.4)	42 (40.8)	48 (48.5)

**Race, n (%)**									

Japanese	48 (100)	50 (100)	50 (100)	51 (100)	53 (100)	49 (100)	99 (100)	103 (100)	99 (100)

**History of allergic reaction, n (%)**									

No	48 (100)	50 (100)	50 (100)	51 (100)	53 (100)	49 (100)	99 (100)	103 (100)	99 (100)

**Complications, n (%)**									

Yes	41 (85.4)	46 (92.0)	41 (82.0)	46 (90.2)	47 (88.7)	42 (85.7)	87 (87.9)	93 (90.3)	83 (83.8)
No	7 (14.6)	4 (8.0)	9 (18.0)	5 (9.8)	6 (11.3)	7 (14.3)	12 (12.1)	10 (9.7)	16 (16.2)

**Medical history, n (%)**									

Yes	0	1 (2.0)	1 (2.0)	2 (3.9)	1 (1.9)	1 (2.0)	2 (2.0)	2 (1.9)	2 (2.0)
No	48 (100)	49 (98.0)	49 (98.0)	49 (96.1)	52 (98.1)	48 (98.0)	97 (98.0)	101 (98.1)	97 (98.0)

**Smoking history, n (%)**									

Yes	2 (4.2)	4 (8.0)	3 (6.0)	5 (9.8)	6 (11.3)	2 (4.1)	7 (7.1)	10 (9.7)	5 (5.1)
No	46 (95.8)	46 (92.0)	47 (94.0)	46 (90.2)	47 (88.7)	47 (95.9)	92 (92.9)	93 (90.3)	94 (94.9)

**History of SARS-CoV-2 infection, n (%)**									

Yes	16 (33.3)	13 (26.0)	15 (30.0)	6 (11.8)	7 (13.2)	6 (12.2)	22 (22.2)	20 (19.4)	21 (21.2)
No	32 (66.7)	37 (74.0)	35 (70.0)	45 (88.2)	46 (86.8)	43 (87.8)	77 (77.8)	83 (80.6)	78 (78.8)

**History of variant vaccine administration, n (%)**									

Yes	28 (58.3)	34 (68.0)	31 (62.0)	41 (80.4)	44 (83.0)	45 (91.8)	69 (69.7)	78 (75.7)	76 (76.8)
No	20 (41.7)	16 (32.0)	19 (38.0)	10 (19.6)	9 (17.0)	4 (8.2)	30 (30.3)	25 (24.3)	23 (23.2)

**Types of previously administered vaccines, n (%)**									

Comirnaty only	16 (33.3)	16 (32.0)	19 (38.0)	25 (49.0)	19 (35.8)	21 (42.9)	41 (41.4)	35 (34.0)	40 (40.4)
Mixed	32 (66.7)	34 (68.0)	31 (62.0)	26 (51.0)	34 (64.2)	28 (57.1)	58 (58.6)	68 (66.0)	59 (59.6)

**Number of SARS-CoV-2 vaccine doses, n (%)**									

2 doses	8 (16.7)	6 (12.0)	7 (14.0)	2 (3.9)	0	0	10 (10.1)	6 (5.8)	7 (7.1)
3–4 doses	33 (68.8)	40 (80.0)	38 (76.0)	15 (29.4)	14 (26.4)	11 (22.4)	48 (48.5)	54 (52.4)	49 (49.5)
5 doses	7 (14.6)	4 (8.0)	5 (10.0)	34 (66.7)	39 (73.6)	38 (77.6)	41 (41.4)	43 (41.7)	43 (43.4)

**Baseline serum IgG antibody titer (AU/mL), n (%)**									

<10,000	21 (43.8)	22 (44.0)	30 (60.0)	24 (47.1)	25 (47.2)	28 (57.1)	45 (45.5)	47 (45.6)	58 (58.6)
≥10,000	27 (56.3)	28 (56.0)	20 (40.0)	27 (52.9)	28 (52.8)	21 (42.9)	54 (54.5)	56 (54.4)	41 (41.4)

**Baseline serum neutralizing antibody titer against virus (Omicron variant BA.5), n (%)**									

< Median (80)	8 (16.7)	15 (30.0)	18 (36.0)	21 (41.2)	22 (41.5)	22 (44.9)	29 (29.3)	37 (35.9)	40 (40.4)
≥ Median (80)	40 (83.3)	35 (70.0)	32 (64.0)	30 (58.8)	31 (58.5)	27 (55.1)	70 (70.7)	66 (64.1)	59 (59.6)

Socioeconomic status was not collected.

IgG, immunoglobulin G; SARS-CoV-2, severe acute respiratory syndrome coronavirus 2; SD, standard deviation.

**Table 2:** Breakdown of participants in the analysis

	Non-Elderly			Elderly			Overall		
	VLPCOV-02		Comirnaty RTU	VLPCOV-02		Comirnaty RTU	VLPCOV-02		Comirnaty RTU
	3 μg	7.5 μg	30 μg	3 μg	7.5 μg	30 μg	3 μg	7.5 μg	30 μg
Full-Analysis-Set	53	55	52	54	55	54	107	110	106
Number of Participants Excluded from Full-Analysis-Set	0	0	0	0	0	0	0	0	0
Per-Protocol-Set	48	50	50	51	53	49	99	103	99
Number of Participants Excluded from Per-Protocol-Set	5	5	2	3	2	5	8	7	7
• COVID-19 was reported as an adverse event and was determined to be a new infection with COVID-19. Visit 03 was not performed within protocol allowance.	0	0	0	2	1	0	2	1	0
• COVID-19 was reported as an adverse event and was determined to be a new infection with COVID-19.	0	0	0	1	0	0	1	0	0
• Positive conversion of SARS-CoV-2 anti-N-IgG antibody titer. COVID-19 was reported as an adverse event and was determined to be a new infection with COVID-19.	0	2	0	0	0	2	0	2	2
• Positive conversion of SARS-CoV-2 anti-N-IgG antibody titer.	3	3	2	0	1	3	3	4	5
• Visit 03 was not performed within protocol allowance.	2	0	0	0	0	0	2	0	0
Safety-Evaluable-Set	53	55	52	54	55	54	107	110	106
Number of Participants Excluded from Safety-Evaluable-Set	0	0	0	0	0	0	0	0	0

Comirnaty RTU = Comirnaty RTU intramuscular (bivalent: wild-type/omicron BA.4-5).

### Safety

**Figure 2:**
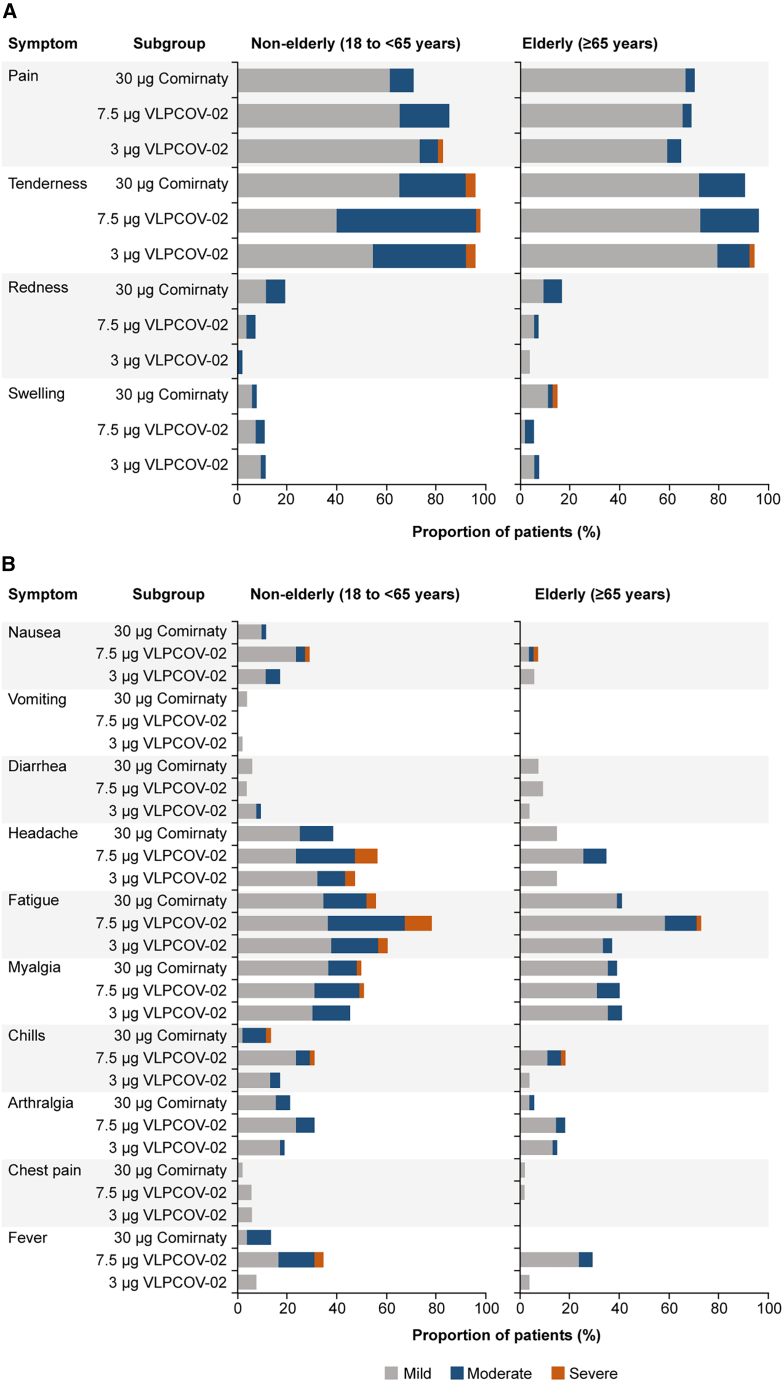
Solicited adverse events reported up to 6 days after study drug administration (day 7) (A) The percentage of solicited local adverse events and their severity reported up to 6 days after study drug administration. (B) The percentage of systemic adverse events and their severity reported up to 6 days after study drug administration. Non-elderly (7.5 μg VLPCOV-02, *n =* 55; 3 μg VLPCOV-02, *n =* 53; 30 μg Comirnaty RTU, *n =* 52). Elderly (7.5 μg VLPCOV-02, *n =* 55; 3 μg VLPCOV-02, *n =* 54; 30 μg Comirnaty RTU, *n =* 54). The severity of solicited adverse events was graded as mild (grey color), moderate (turquoise color), or severe (orange color), assessed using Common Terminology Criteria in Solid Tumors version 5.0 and per United States Food and Drug Administration guidance (Toxicity Grading Scale for Healthy Adult and Adolescent Volunteers Enrolled in Preventive Clinical Trials).

The safety profile of VLPCOV-02 was consistent with previous reports, and no major concerns were reported. Following booster vaccination with either dose of VLPCOV-02, the majority of solicited adverse events reported were mild-to-moderate in severity (Figure 2). Overall incidence of fatigue, headache, fever, chills, arthralgia, and nausea was higher in the 7.5 μg VLPCOV-02 cohort compared with the 3 μg cohort, and similar in the 3 μg VLPCOV-02 cohort and 30 μg Comirnaty RTU Wild type/Omicron BA.4-5 cohort. Induration was reported in 29 (27.1%), 34 (30.9%), and 41 (38.7%) of the 3 μg VLPCOV-02, 7.5 μg VLPCOV-02, and 30 μg Comirnaty RTU cohorts, respectively.

In this study, ECGs were performed at baseline to ensure that no abnormalities were present prior to immunization. Nine participants (three from the non-elderly 3 μg group, three from the non-elderly 7.5 μg group, one from the elderly 7.5 μg group, and one from each non-elderly and elderly 30 μg Comirnaty RTU group) reported mild chest pain post immunization. For these participants, a 12-lead ECG, blood biochemistry, and cardiac troponin I tests were performed per study protocol. Some cases showed mild increases in CRP and decreases in CK, but no signs of myocarditis or pericarditis were detected.

### VLPCOV-02 elicits robust, durable antibody responses against severe acute respiratory syndrome coronavirus 2 variants

**Figure 3:**
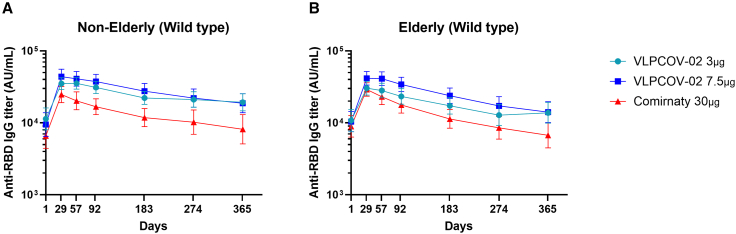
Anti-RBD IgG responses Serum IgG antibody titers against SARS-CoV-2 RBD protein (Wild type) for non-elderly (18 to <65 years, left side) and elderly (≥65 years, right side) participants. Logarithmic values are reported as geometric mean titers. Bars indicate 95% confidence intervals. The upper limit of detection is 160,000, and the lower limit of detection is 6.8 AU/mL. Non-elderly (7.5 μg VLPCOV-02, *n =* 50; 3 μg VLPCOV-02, *n =* 48; 30 μg Comirnaty RTU, *n =* 50). Elderly (7.5 μg VLPCOV-02, *n =* 53; 3 μg VLPCOV-02, *n =* 51; 30 μg Comirnaty RTU, *n =* 49), also see Table S1.

The evaluation of circulating anti-RBD IgG titers showed a booster effect with both doses of VLPCOV-02, and in both elderly and non-elderly cohorts (Figure 3). Overall, the geometric mean titers (GMTs) at week 4 were 32,572.6 (95% confidence interval [CI]: 28,192.9–37,632.6) for 3 μg VLPCOV-02, 42,751.4 (95% CI: 36,512.2–50,056.7) for 7.5 μg VLPCOV-02, and 26,840.4 (95% CI: 22,717.1–31,712.0) for 30 μg Comirnaty RTU. Importantly, IgG titers remained high at 52 weeks post-vaccination with 3 μg VLPCOV-02 (15,933.0; 95% CI 12,773.1–19,874.4) and 7.5 μg VLPCOV-02 (16,148.8; 95% CI: 12,900.0–20,215.9), and continued to be higher than IgG titers measured following vaccination with 30 μg Comirnaty RTU (7,229.8; 95% CI: 5,369.1–9,735.4). Serum neutralizing antibody titers against SARS-CoV-2 RBD protein are shown in Table S1.

**Figure 4:**
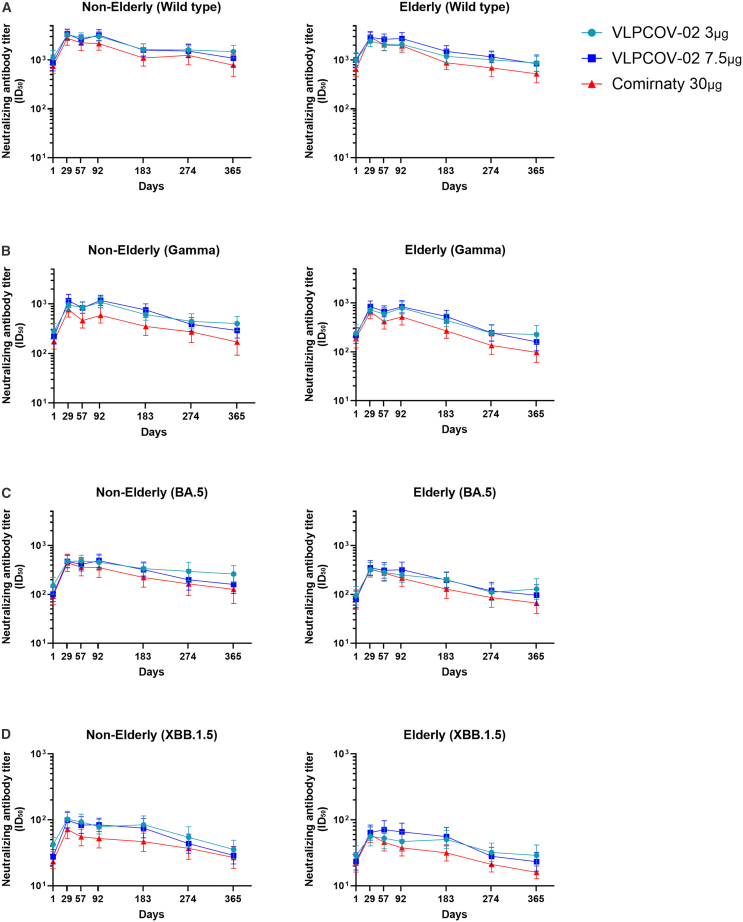
Neutralizing antibody responses against SARS-CoV-2 variants Serum neutralizing antibody titers against (A) Wild type, (B) Gamma, (C) Omicron BA.5, and (D) Omicron XBB.1.5 variants for non-elderly (18 to <65 years, left side) and elderly (≥65 years, right side) participants. Logarithmic values are reported as geometric mean titers. Bars indicate 95% confidence intervals. The lower limit of detection is 20 ID50. Non-elderly (7.5 μg VLPCOV-02, *n =* 50; 3 μg VLPCOV-02, *n =* 48; 30 μg Comirnaty RTU, *n =* 50). Elderly (7.5 μg VLPCOV-02, *n =* 53; 3 μg VLPCOV-02, *n =* 51; 30 μg Comirnaty RTU, *n =* 49), also see Table S2.

Neutralizing antibody titers against all variants of SARS-CoV-2 tested were induced by both doses of VLPCOV-02 in elderly and non-elderly participants, to levels comparable with 30 μg Comirnaty RTU. At week 4, overall serum neutralizing antibody GMTs against the Omicron BA.5 strain were 386.6 (95% CI: 309.2–483.3) for 3 μg VLPCOV-02, 405.0 (95% CI: 322.9–507.9) for 7.5 μg VLPCOV-02, and 381.9 (95% CI: 296.2–492.4) for 30 μg Comirnaty RTU. Notably, at week 52, GMTs were higher among participants receiving 3 μg VLPCOV-02 vs. 30 μg Comirnaty RTU, with an estimated GMT ratio of 2.0 (95% CI: 1.2–3.4) for 3 μg VLPCOV-02 and 1.4 (95% CI: 0.9–2.4) for 7.5 μg VLPCOV-02. For the Omicron XBB.1.5 strain, overall GMTs at week 4 were 75.1 (95% CI: 60.0–94.0) for 3 μg VLPCOV-02, 78.9 (95% CI: 65.0–95.8) for 7.5 μg VLPCOV-02, and 65.2 (95% CI: 53.1–80.0) for 30 μg Comirnaty RTU. The estimated ratio of GMT to 30 μg Comirnaty RTU was consistently ≥1 across all time points for neutralizing titers against wild-type, Gamma, Omicron BA.5, and Omicron XBB.1.5. Neutralizing titers for all strains are shown in Figure 4 and Table S2.

Serum neutralizing antibody titers against pseudovirus Omicron BA.5 at week 4 were 1,829.7 (95% CI: 1,414.2–2,367.4) for 3 μg VLPCOV-02, 1,713.5 (95% CI: 1,335.3–2,198.7) for 7.5 μg VLPCOV-02, and 1,879.3 (95% CI: 1,402.3–2,518.4) for 30 μg Comirnaty RTU. Notably, at week 52, GMTs were higher among participants receiving 3 μg VLPCOV-02 vs. 30 μg Comirnaty RTU (739.3 [95% CI: 482.3–1,133.2] for 3 μg VLPCOV-02, 481.0 [95% CI: 321.0–720.7] for 7.5 μg VLPCOV-02, and 288.1 [95% CI: 175.3–473.6] for 30 μg Comirnaty RTU). Serum neutralizing antibody titers against pseudovirus are shown in Figure S1 and Table S3.

### VLPCOV-02 induces strong T cell responses across age groups

**Figure 5:**
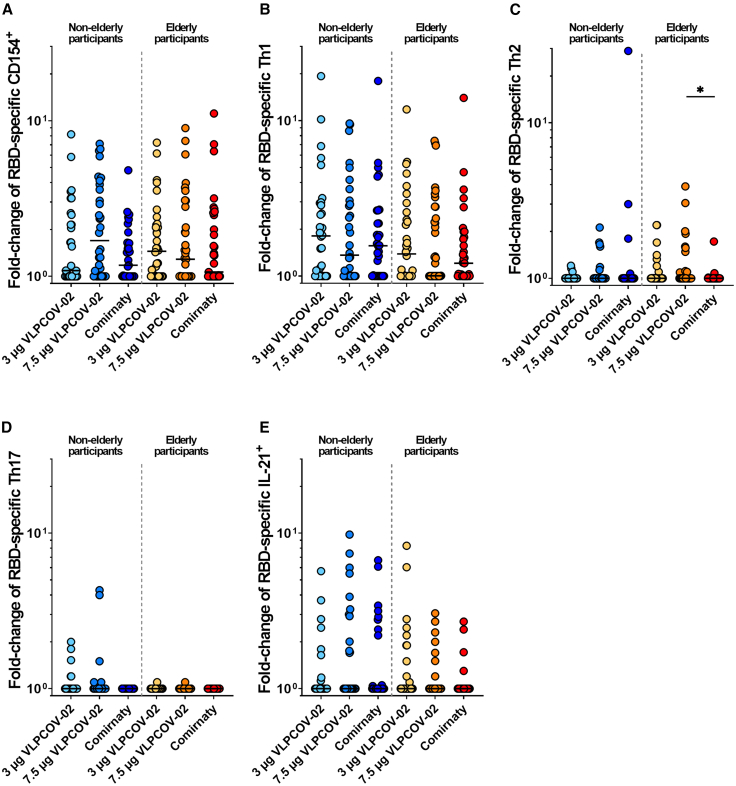
CD4^+^ T cell responses against RBD Flow cytometric analysis was performed to analyze RBD-specific T cell responses. Responses to 3 μg or 7.5 μg of VLPCOV-02 or 30 μg of Comirnaty RTU are shown as fold-change from baseline (day 1) to week 4 (day 29) for each cohort. Panel A shows the activated CD4^+^ T cells, which were characterized by the expression of CD154^+^. Panel B shows the response in CD4^+^ Th1 cells, which was characterized by the expression of IL-2, TNF, and/or IFN-γ. Panel C shows the response in CD4^+^ Th2 cells, which was characterized by the expression of IL-4 and/or IL-13. Panel D shows the response in CD4^+^ Th17 cells, which was characterized by the expression of IL-17. Panel E shows the CD4^+^ IL-21^+^ cells, which were characterized by the expression of IL-21. The horizontal bars indicate median values. *p* values (two-sided) were calculated using the Mann-Whitney *U*-test. Non-elderly (7.5 μg VLPCOV-02, *n =* 32; 3 μg VLPCOV-02, *n =* 32; 30 μg Comirnaty RTU, *n =* 31). Elderly (7.5 μg VLPCOV-02, *n =* 37; 3 μg VLPCOV-02, *n =* 34; 30 μg Comirnaty RTU, *n =* 35). IFN, interferon; IL, interleukin; TNF, tumor necrosis factor, also see Figure S2.

CD4^+^ T cell responses were induced by VLPCOV-02 booster vaccination in both non-elderly and elderly participants in response to the RBD-specific peptide pool. The fold-change in the frequency of CD154-positive CD4^+^ T cells against RBD tended to be higher in the VLPCOV-02 vaccination group compared to the Comirnaty RTU vaccination group (among non-elderly, median 1.70 in 7.5 μg of VLPCOV-02 vs. 1.18 in 30 μg of Comirnaty RTU fold-change [*p* = 0.0604]; among elderly, median 1.29 in 7.5 μg of VLPCOV-02 vs. 1.14 in 30 μg of Comirnaty RTU fold-change [*p* = 0.911]). Across all VLPCOV-02 dose groups, the responses were predominantly Th1-skewed (characterized by IFN-γ, IL-2, and/or tumor necrosis factor [TNF] production), and IL-21-producing RBD-specific CD4^+^ T cells were also detected (Figures 5 and S2). The Th17 response (characterized by IL-17 production) was minimal, whereas in elderly participants, a mild Th2 response (characterized by IL-4 and/or IL-13 production) was observed (*p* = 0.0225 between 7.5 μg of VLPCOV-02 and 30 μg of Comirnaty RTU). Regarding responses to the spike-specific peptide pool, VLPCOV-02 induced Th1, Th2, and IL-21 responses comparable to those induced by Comirnaty, with no significant differences observed between the two vaccines (Figures S3 and S4).

**Figure 6:**
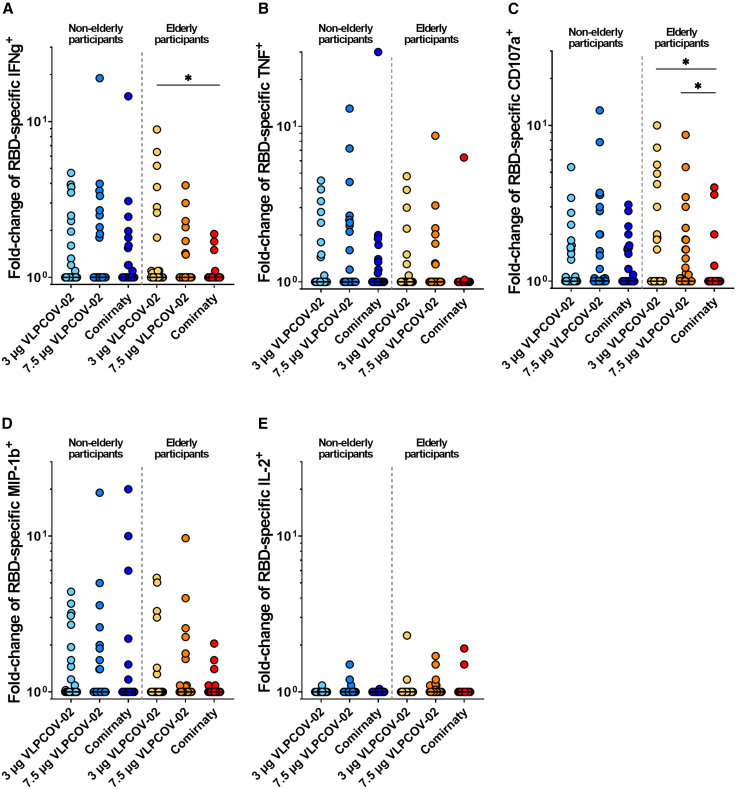
CD8^+^ T cell responses against RBD Flow cytometric analysis was performed to analyze RBD-specific T cells. Responses to VLPCOV-02 or Comirnaty RTU are shown as fold change from baseline (day 1) to week 4 (day 29) for each cohort. RBD-specific CD8^+^ T cells were determined based on the expression of (A) IFN-γ, (B) TNF, (C) CD107a, (D) MIP-1β, or (E) IL-2, respectively. *p* values (two-sided) were calculated using the Mann-Whitney *U*-test. Non-elderly (7.5 μg VLPCOV-02, *n =* 32; 3 μg VLPCOV-02, *n =* 32; 30 μg Comirnaty RTU, *n =* 31). Elderly (7.5 μg VLPCOV-02, *n =* 37; 3 μg VLPCOV-02, *n =* 34; 30 μg Comirnaty RTU, *n =* 35). IFN, interferon; IL, interleukin; RBD, receptor-binding domain; TNF, tumor necrosis factor, also see Figure S5.

For RBD-specific CD8^+^ T cell responses, VLPCOV-02 induced RBD-specific CD8^+^ T cells expressing IFN-γ, TNF, or CD107a production higher than those induced by 30 μg of Comirnaty RTU (Figures 6 and S5). Notably, in elderly participants, significant differences were observed in IFN-γ and CD107a production (IFN-γ: *p* = 0.0234 between 3 μg of VLPCOV-02 and 30 μg of Comirnaty RTU in elderly participants; CD107a: *p* = 0.0429 between 3 μg of VLPCOV-02 and 30 μg of Comirnaty RTU and *p* = 0.0128 between 7.5 μg of VLPCOV-02 and 30 μg of Comirnaty RTU in elderly participants). Similarly, in response to the spike-specific peptide pool, VLPCOV-02 induced CD8^+^ T cell responses that differed from those elicited by Comirnaty RTU. The pattern of these responses was consistent with observations seen in the RBD-specific CD8^+^ T cell responses (IFN-γ: *p* = 0.3447 between 3 μg of VLPCOV-02 and 30 μg Comirnaty RTU in elderly participants; CD107a: *p* = 0.1917 between 7.5 μg of VLPCOV-02 and 30 μg of Comirnaty RTU in elderly participants). Similarly, in response to the spike-specific peptide pool, VLPCOV-02 showed CD8^+^ T cell responses that were numerically higher, comparable to the RBD-specific CD8^+^ T cell responses, without reaching statistical significance when compared to Comirnaty RTU (Figures S6 and S7). No notable differences were observed in MIP-1β^+^ or IL-2^+^ CD8^+^ T cell responses between the VLPCOV-02 and Comirnaty RTU groups.

## Discussion

Part 1 of the phase 1/2 study demonstrated that the 5 mC modification was compatible without compromising immunogenicity, with VLPCOV-02 booster vaccination inducing robust IgG responses and a reduction in systemic adverse events experienced when compared to results previously reported for VLPCOV-01. In the results presented here from part 2 of the study, which evaluated VLPCOV-02 in a head-to-head comparison with the bivalent variant-adapted Comirnaty RTU vaccine, the safety profiles were similar, and no new safety signals were identified. The main objective of part 2 of this study was to ascertain the optimal dose of VLPCOV-02 for continued clinical development. Based on the safety and immunogenicity results presented here, this dose was determined to be 3 μg, which is one-tenth of the comparator mRNA vaccine dose. The ability to induce durable immune responses with a low-dose vaccine such as VLPCOV-02 has important implications for the scalability of COVID-19 vaccine development, whereby the manufacturing time and distribution of variant-adapted vaccines in response to the emergence of new variants can be accelerated.

The safety and reactogenicity of the Comirnaty original and variant-adapted vaccines have been studied extensively, including through clinical development, post-marketing surveillance, and real-world data. The vaccines have been generally well tolerated, and their safety and reactogenicity profiles have remained consistent, with most adverse reactions being transient and managed with medications or preventative measures.^12^

Previously, in a phase 1 study of VLPCOV-01, neutralizing antibody titers to multiple SARS-CoV-2 variants were demonstrated up to 26 weeks following VLPCOV-01 booster vaccination, and a strong correlation of anti-SARS-CoV-2 IgG titers with neutralization titers was observed, suggesting that IgG titers may be used as a predictor of neutralizing activity.^5^ In this study, we observed durable anti-RBD IgG titers following VLPCOV-02 booster vaccination, and neutralizing titers to multiple strains, including XBB.1.5, were observed up to 52 weeks. VLPCOV-02 also induced Gamma RBD- and XBB-specific CD4^+^ and CD8^+^ T cell responses that were comparable to Comirnaty RTU, suggesting that the induction of humoral and cellular immune responses in both elderly and non-elderly cohorts at doses of 3 μg and 7.5 μg VLPCOV-02 were achieved.

COVID-19 mRNA vaccines have demonstrated a decline in neutralizing antibody titers over 3–6 months, necessitating the need for booster vaccinations.^13^^,^^14^^,^^15^ VLPCOV-02 induced humoral immune responses against multiple circulating SARS-CoV-2 variants, despite the anchored RBD being derived from the Gamma variant. In addition to being able to modify the antigenic domain of vaccines to address emerging variants of concern, as was done in the development of VLPCOV-02, it is also important to be able to induce immune responses to other subvariants. The comparator vaccine in this study, Comirnaty RTU (Bivalent: Wild type/Omicron BA.4-5), is a variant-adapted vaccine for Omicron subvariants BA.4-5.

While some studies have proposed potential correlates of protection based on neutralizing antibody titers, it is important to note that the values obtained from different neutralization assays can vary significantly. As such, there is no universally accepted threshold for protection, and reported values depend on the specific assay used. Consequently, an absolute threshold for neutralizing antibodies has not been established in the literature, and comparisons across studies remain challenging.

The impact of VLPCOV-02 on the innate immune response in the early post-vaccination period was not assessed in this study. However, we recognize the significance of this information in elucidating the mechanistic properties of the 5 mC vaccine, particularly its ability to maintain immunogenicity while reducing reactogenicity. A recent preclinical study, currently under review for publication, describes the impact of introducing 5 mC to saRNA to further contribute to our understanding of the 5 mC vaccine’s mechanism of action and its potential advantages in balancing immunogenicity and reactogenicity.^6^

In September and October 2023, the updated Moderna and Pfizer-BioNTech mRNA vaccines against COVID-19 containing the SARS-CoV-2 Omicron XBB.1.5 subvariant and the updated Novavax adjuvanted vaccine against COVID-19 containing the spike protein from the XBB.1.5 subvariant were authorized by the Food and Drug Administration, respectively. Following approval in the United States, rapid changes to the dominant circulating variants were observed, whereby the prevalence of the XBB.1.5 subvariant was greatly reduced. Nevertheless, the XBB.1.5 vaccines demonstrated effectiveness against other, more prevalent, Omicron subvariants, although less so against subvariant JN.1.^16^ The Comirnaty RTU intramuscular injection (monovalent: Omicron strain XBB.1.5) was also approved in September 2023 by the Japanese Ministry of Health, Labor and Welfare. For the continued clinical development of VLPCOV-02, the RBD has been updated to the XBB.1.5 subvariant, and the resultant vaccine, VLPCOV-04, is currently being evaluated at a dose of 3 μg in a phase 3 trial.

Part 2 of this phase 1/2 study demonstrates that VLPCOV-02 has an acceptable safety and immunogenicity profile among adults previously vaccinated against SARS-CoV-2. The incorporation of a modified 5 mC base improves the safety profile of the saRNA platform without compromising immunogenicity, supporting further development of this vaccine as a booster in response to circulating SARS-CoV-2 variants of concern.

### Limitations of the study

Limitations of this study include the relatively small sample size and the fact that the study was limited to the Japanese population; the number of participants will be further expanded in the ongoing phase 3 study.

While our study was designed to include a comparison between the investigational product and the VLPCOV-02 groups, comparisons between age subgroups were not conducted. This limits our ability to conclude the vaccine’s differential effects across elderly and non-elderly cohorts, but will be considered for future studies to provide a more comprehensive understanding of the vaccine’s performance across age groups.

## Resource availability

### Lead contact

Requests for further information and resources should be directed to and will be fulfilled by the lead contact, Wataru Akahata (wakahata@vlptherapeutics.com).

### Materials availability

This study did not generate new unique reagents.

### Data and code availability

• Data reported in this article will be shared by the lead contact upon request.
• This article does not report original code.
• Any additional information required to reanalyze the data reported in this article is available from the lead contact upon request.

## Acknowledgments

VLP Therapeutics Japan, Inc. served as the trial sponsor and was responsible for the design and conduct of the trial, for the collection, analysis, and interpretation of the data, and for the writing of the article. This study was supported by 10.13039/100009619AMED, Japan, under Grant Number JP21nf0101627. Medical writing support was provided by Emily Feist, PhD (10.13039/100016751Parexel International), and was funded by VLP Therapeutics Japan, Inc. All participants provided written informed consent to participate before enrollment. We thank all the members of the Laboratory of Precision Immunology, Center for Intractable Diseases and ImmunoGenomics and National Institutes of Biomedical Innovation, Health and Nutrition, Osaka, Japan, for PBMC preparation and technical support. We also express our deep thanks to Shoko Nishimori and Rumi Oono at VLP Therapeutics Japan, Inc for clinical operations support.

## Author contributions

M.A., D.K., K.K., K.M., T.S., S.S., J.F.S., N.S., T.Y., and W.A. conceived, designed, and coordinated the study. M.A., D.K., K.K., A.N., T.N., Y.S., S.S., and N.S. prepared and executed the clinical study. K.M., M.A., D.K., K.K., A.N., T.N., T.Y., and W.A. contributed to the data validation, analysis, writing, and revision of the article. All authors read and approved the article, had full access to all of the data in the study, and had final responsibility for the decision to submit for publication.

## Declaration of interests

M.A., D.K., K.K., Y.S., T.S., and N.S. are employees of VLP Therapeutics Japan, Inc.; K.M. is an employee of VLP Therapeutics, Inc.; W.A. is a board member, an employee, and holds stocks in VLP Therapeutics, Inc. and is a management board member of VLP Therapeutics Japan, Inc.; J.F.S. is an employee and holds stocks in VLP Therapeutics, Inc.; S.S. received a consultation fee from VLP Therapeutics Japan, Inc. for medical advice and consultation on clinical trial design. W.A. and J.F.S. are inventors on a related vaccine patent. The remaining authors declare no competing interests.

## STAR★Methods

### Key resources table

**Table:** 

REAGENT or RESOURCE	SOURCE	IDENTIFIER
**Antibodies**		

Mouse anti-human CD154-FITC (clone: TRAP1)	BD Biosciences	cat# 55569; RRID: AB_396049
Mouse anti-human CD3-BUV615 (clone: SP34-2)	BD Biosciences	cat# 751249; RRID: AB_2875266
Mouse anti-human CD4-PE-Cy5.5 (clone: S3.5)	Thermo Fisher Scientific	cat# MHCD0418; RRID: AB_10376013
Mouse anti-human CD8-BUV563 (clone: RPA-T8)	BD Biosciences	cat# 612914; RRID: AB_2870200
Mouse anti-human CD27-PE-Cy5 (clone: 1A4CD27)	Beckman Coulter	cat# 6607107; RRID: AB_10641617
Mouse anti-human CD45RO-BUV805 (clone: UCHL1)	BD Biosciences	cat# 748367; RRID: AB_2872786
Mouse anti-human IFN-γ-BV786 (clone: 4S.B3)	BioLegend	cat# 502542; RRID: AB_2563882
Mouse anti-human TNF-BV650 (clone: MAb11)	BioLegend	cat# 502938; RRID: AB_2562741
Rat anti-human IL-13-BV421 (clone: JES10-5A2)	BD Biosciences	cat# 563580; RRID: AB_2738290
Mouse anti-human IL-21-Ax647 (clone: 3A3-N21)	BD Biosciences	cat# 560493; RRID: AB_1645421
Mouse anti-human IL-4-PE-Cy7 (clone:8D4-8)	BD Biosciences	cat# 560672; RRID: AB_1727547
Mouse anti-human IL-17A-BV605 (clone: BL168)	BioLegend	cat# 512326; RRID: AB_2563887
Rat anti-human IL-2-BUV737 (clone: MQ1-17H12)	BD Biosciences	cat# 612836; RRID: AB_2870158
Mouse anti-human CD107A-BV711 (clone: H4A3)	BioLegend	cat# 328640; RRID: AB_2565840
Mouse anti-human MIP1β-Alexa700 (clone: D21-1351)	BD Biosciences	cat# 561278; RRID: AB_10612008

**Biological samples**		

Human PBMCs	This study	This study
Human serum	This study	This study

**Chemicals, peptides, and recombinant proteins**		

Benzonase Nuclease, Purity >90%	Merck Millipore	cat# 70746
BD GolgiPlug	BD Biosciences	cat# 555029
BD GolgiStop	BD Biosciences	cat# 554724
LIVE/DEADTM Fixable Blue Dead Cell Stain Kit	Thermo Fisher Scientific	cat# L23105
Cytofix/Cytoperm kit	BD Biosciences	cat# 554714; RRID: AB_2869008

**Experimental models: Cell lines**		

Vero cells	ATCC	CCL81

**Software and algorithms**		

FlowJo 10.10.0	BD Biosciences	https://www.flowjo.com/
GraphPadPrism version 9	GraphPad Software, Inc.	https://www.graphpad.com

### Experimental model and study participant details

#### Study design and population

This trial was a two-part phase 1/2 study. Results from part 1, a phase 1 dose-escalation, open-label study to assess the safety, tolerability, and immunogenicity of VLPCOV-02 as a single booster dose, have been published.^7^ Part 2 is a phase 2 multicenter, randomized, active comparator-controlled, observer-blinded study to determine the recommended booster dose of VLPCOV-02. Before the start of the study, the Institutional Review Board reviewed the appropriateness of conducting this study based on the submitted Protocol, Investigator's Brochure, informed consent form, and other related documents, and gave their approval. Ethics committee approval was obtained by the Institutional Review Board of the Medical Corporation Heishinkai OPHAC Hospital (No. 1160PB). The Principal Investigator or Sub-investigators handed the informed consent form and written information to subjects and explained them to subjects so that they could correctly understand the details of the study. After the subjects fully understood the details of the study, voluntary consent to participate in the study was obtained in writing from the subjects before all the observations/tests, etc. described in the Protocol were conducted. In the Part 2, of the 166 non-elderly and 170 elderly subjects who gave written informed consent to participate in the study, 160 non-elderly and 163 elderly subjects for whom eligibility was confirmed during screening were randomized.

Participant size was determined using EMA’s guidance on influenza vaccine (CPMP/BWP/214/96). Participants were randomized to three treatment groups: two investigational drug groups and one comparator group. Each group comprised a non-elderly cohort (18 to <65 years) and an elderly cohort (≥65 years). Participants were randomly assigned to each study drug group within each age cohort and stratified according to presence or absence of a history of SARS-CoV-2 infection, number of vaccinations against SARS-CoV-2, and study site. The planned number of participants, as set out in the study protocol, was 300, which was deemed the minimal number of participants required to evaluate safety and immunogenicity of VLPCOV-02; this is not a setting based on the statistical considerations.

Participants were registered and randomized using the Medidata Rave randomization and trial supply management (RTSM) system. Randomization was stratified by prior SARS-CoV-2 infection status, number of previous SARS-CoV-2 vaccinations (≤2 vs ≥3), and study site.

Eligible participants were healthy Japanese adults aged ≥18 years who had received the two-dose primary vaccination series with the same COVID-19 uridine-modified RNA vaccine or a booster vaccination with an mRNA vaccine (any kind, any number, and including bivalent mRNA vaccines) ≥6 months previously. Key exclusion criteria were a history of SARS-CoV-2 infection ≤6 months prior to study day 1; persistent symptoms of any kind following either SARS-CoV-2 infection or previous vaccination against COVID-19; a history or presence of a serious cardiovascular, hematologic, respiratory, hepatic, renal, gastrointestinal, and/or neuropsychiatric disease; and presence or known history of a disease or previous/planned receipt of any agent or therapy that could affect immunogenicity assessments. Pregnant and lactating females were also excluded from study entry. All participants provided written informed consent before enrollment. Full eligibility criteria are described in the study protocol. Sex was not included as a predefined stratification factor in this study. However, the distribution of male and female participants was well balanced across treatment groups (Table 11.2-2), minimizing the potential impact of sex-related bias on the study outcomes.

#### Cell lines

The Vero cells (kidney epithelial cells extracted from an African green monkey) were authenticated by ATCC using isoenzyme analysis and confirmed mycoplasma negative. Vero cells are maintained in DMEM supplemented with 10% FBS at 37°C. Peripheral blood mononuclear cells (PBMCs) obtained from participants were maintained in RPMI medium containing 10% FBS at 37°C.

### Method details

#### Trial procedures

Based on the safety and immunogenicity results following study drug inoculation in part 1, two dose levels of VLPCOV-02 were investigated (3 μg and 7.5 μg) and compared with 30 μg Comirnaty RTU intramuscular injection (Bivalent: Wild type/Omicron BA.4-5).

A medical doctor or nurse administered the prespecified doses as an intramuscular injection to the upper-arm deltoid muscle of each participant, who were then carefully monitored for up to 30 minutes after administration, including medical examination, blood pressure, pulse rate, body temperature, and 12-lead electrocardiogram (ECG). Follow-up visits were scheduled from day 8 up to week 52 to review any changes in concomitant medications, collect vital signs, review safety assessments, and obtain blood samples for immunogenicity analyses.

#### Safety assessments

The primary safety endpoints were incidence and severity of solicited local (pain, tenderness, redness, induration, and swelling) and systemic (nausea, vomiting, diarrhea, headache, fatigue, myalgia, chills, arthralgia, chest pain, and fever) adverse events that occurred up to 1 week after study drug administration and any adverse events that occurred up to 4 weeks after study drug administration. Secondary safety endpoints were the incidence of serious adverse events, COVID-19 infection, and adverse events leading to study discontinuation that occurred up to 52 weeks after study drug administration or until study discontinuation. If symptoms of suspected myocarditis/pericarditis were recorded, 12-lead ECG and blood biochemistry (creatine kinase, C-reactive protein, cardiac troponin I) were conducted, with diagnostic imaging as needed.

#### Immunogenicity assessments

Primary immunogenicity endpoints were GMT and seroresponse rate of serum neutralizing antibody titers against live virus (Omicron BA.5) at 4 weeks after study drug administration. Secondary endpoints included measurement of IgG titers against SARS-CoV-2 RBD protein and serum neutralizing antibody titers against pseudovirus (Omicron BA.5). Exploratory endpoints included evaluation of the serum neutralizing antibody titers against live virus and pseudovirus of Wild type, Gamma, and Omicron XBB.1.5 variants, and functional assessments of S antigen-specific T- and B-cells derived from PBMCs.

The immunogenicity assessments performed have been previously described.^5^ Briefly, serum IgG titers were quantified in samples analyzed using the SARS-CoV-2 RBD IgG II Quant assay, which detects IgG antibodies to the RBD of the SARS-CoV-2 spike protein, according to the manufacturer’s instructions (Abbott Laboratories). The pseudovirus neutralization assays were conducted using Vero cells as previously described.^5^ Briefly, serum samples were activated, diluted, and mixed with pseudotyped virus, and assessed with the Luciferase Assay System (Promega). Luciferase activity of the cells was measured by Synergy LC (Bio Tek) and analyzed using GraphPad Prism 8. Pseudovirus neutralizing antibody titers were calculated as 50% inhibitory dilution.

For analyzing antigen-specific T-cells, flow cytometric analysis was performed. The cell staining protocol has been previously described.^5^ Briefly, PBMCs obtained from participants were incubated in 200 μL Roswell Park Memorial Institute medium containing 10% FBS with or without peptides (17-mers overlapping by 10 residues) corresponding to the RBD region or the full SARS-CoV-2 spike region, at a final concentration of 2 μg/mL of each peptide in the presence of anti-CD107a (H4A3). Thereafter, 0.2 μL BD GolgiPlug and 0.14 μL BD GolgiStop (both from BD Biosciences) were added to the cells and the cells were incubated for 5.5 h. The cells were then stained using the LIVE/DEAD™ Fixable Blue Dead Cell Stain Kit (Thermo Fisher Scientific) and stained with anti-CD3 (SP34-2), anti-CD4 (S3.5), anti-CD8 (RPA-T8), anti-CD27 (1A4CD27), and anti-CD45RO (UCHL1) antibodies. After fixation and permeabilization using the Cytofix/Cytoperm kit (BD Biosciences), the cells were stained with anti-IFN-γ (4S.B3), anti-TNF (MAb11), anti-CD154 (TRAP1), anti-IL-13 (JES10-5A2), anti-IL-21 (3A3-N21), anti-IL-4 (8D4-8), anti-IL-17A (BL168), anti-MIP1β (D21-1351), and anti-IL-2 (MQ1-17H12) antibodies. After washing, the cells were fixed with 1% paraformaldehyde and analyzed using a FACSymphony A5 instrument equipped with five lasers (BD Biosciences). Data were analyzed using the FlowJo software version 10.10.0 (BD Biosciences). After gating live single T-cells, based on forward scatter area and height, side scatter area, live/dead cell exclusion, and CD3 staining, we separated the PBMCs into CD4^+^ and CD8^+^ T-cells. Subsequently, CD4^+^ and CD8^+^ T-cells were further divided into memory phenotypes based on the expression of CD27 and CD45RO. For RBD-specific CD4^+^ T-cells, memory cells were gated based on the expression of CD154. We defined CD154^+^ CD4^+^ T-cells expressing IFN-γ, TNF, or IL-2 as Th1 cells, expressing IL-4 or IL-13 as Th2 cells, expressing IL-17 as Th17 cells, and expressing IL-21 as IL-21^+^ cells. For CD8^+^ T-cell responses, antigen-specific CD8^+^ T-cells were determined based on the expression of CD107a, IFN-γ, TNF, IL-2, and MIP-1β. The gating strategy used is shown in Figure S8. The background of frequencies of cytokine production (measured in dimethyl sulfoxide control) were subtracted.

PBMC samples for T-cell analysis were collected only at two of the three study sites (Osaka Clinical Hospital and OCROM Clinic) where PBMC sampling was implemented. Accordingly, the number of participants included in the T-cell analyses reflects the per-protocol subset enrolled at these two sites.

### Quantification and statistical analysis

The data cutoff date for this analysis, which was not prespecified, was July 31, 2024. Safety analyses were analyzed descriptively and are presented as numbers and percentages of participants who experienced adverse events by severity.

Serum neutralizing antibody titers against live virus, serum neutralizing antibody titers against pseudovirus, and serum IgG titers were assessed at weeks 4, 26, and 52 after study drug inoculation, and GMT and related two-sided 95% CIs were calculated by group at each time point. For the primary endpoints, changes in values between pre- and post-vaccination in the investigational drug groups were compared to those in the comparator group.

Statistical analyses for T cell immunogenicity data shown in Figures S2–S7 were performed using a two-tailed Mann–Whitney U test. All statistical analyses were conducted using GraphPad Prism (version 10.5.0), and exact P values are reported in the results section.

### Additional resources

The study protocol and any amendments were approved by Nobuaki Sato or Masayuki Aboshi (VLP Therapeutics Japan, Inc.) and complied with relevant regulatory requirements. This trial is registered with the Japan Registry of Clinical Trials (jRCT2051230005).

Description: https://jrct.mhlw.go.jp/latest-detail/jRCT2051230005.

## Supplemental information

Document S1. Figures S1–S8

Table S1. Serum neutralizing antibody titers against SARS-CoV-2 RBD protein, related to Figure 3

Table S2. Serum neutralizing antibody titers against SARS-CoV-2 variants, related to Figure 4

Table S3. Serum neutralizing antibody titers against SARS-CoV-2 pseudoviruses, related to Results
