# Supplementary material for: Safety, immunogenicity, and optimal dosing of VLPCOV-02, a SARS-CoV-2 saRNA vaccine with modified 5-methylcytosine base
Source: iScience. 2026 Jan 21;29(2):114766. doi: 10.1016/j.isci.2026.114766 (PMC12907056; doi:10.1016/j.isci.2026.114766)
Supplement: Document S1. Figures S1–S8 [file mmc1.pdf]

iScience, Volume *29*

Supplemental information

Safety, immunogenicity, and optimal dosing of VLPCOV-02, a SARS-CoV-2 saRNA vaccine with modified 5-methylcytosine base

Masayuki Aboshi, Daisuke Kawakami, Kaoru Kono, Ayae Nishiyama, Takuto Nogimori, Yuko Sunada, Kenta Matsuda, Takashi Sekida, Shigeru Suga, Jonathan F. Smith, Nobuaki Sato, Takuya Yamamoto, and Wataru Akahata

SUPPLEMENTARY

Figure S1. Neutralizing antibody responses against SARS-CoV-2 pseudoviruses, related to Results

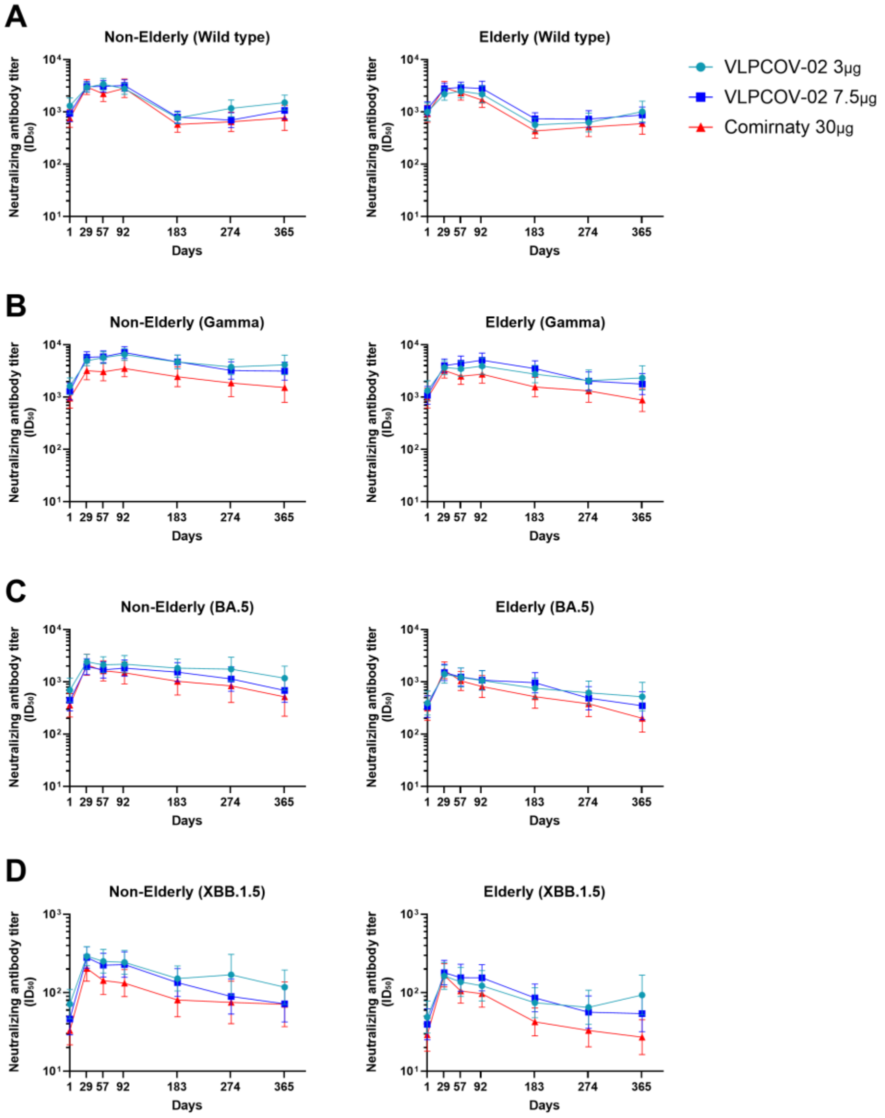

Serum neutralizing antibody titers against A) Wild type, B) Gamma, C) Omicron BA.5, and D) Omicron XBB.1.5 pseudoviruses for non-elderly (18 to <65 years, left side: 7.5 μg VLPCOV-02, *n* = 50; 3 μg VLPCOV-02, *n* = 48; 30 μg Comirnaty RTU, *n* = 50) and elderly (≥65 years, right side: 7.5 μg VLPCOV-02, *n* = 53; 3 μg VLPCOV-02, *n* = 51; 30 μg Comirnaty RTU, *n* = 49) participants. Logarithmic values are reported as geometric mean titers. Bars indicate 95% CIs. The lower limit of detection was 10 ID_{50}.

Figure S2. Frequency of CD4⁺ T-cell responses against RBD gamma, related to Figure 5

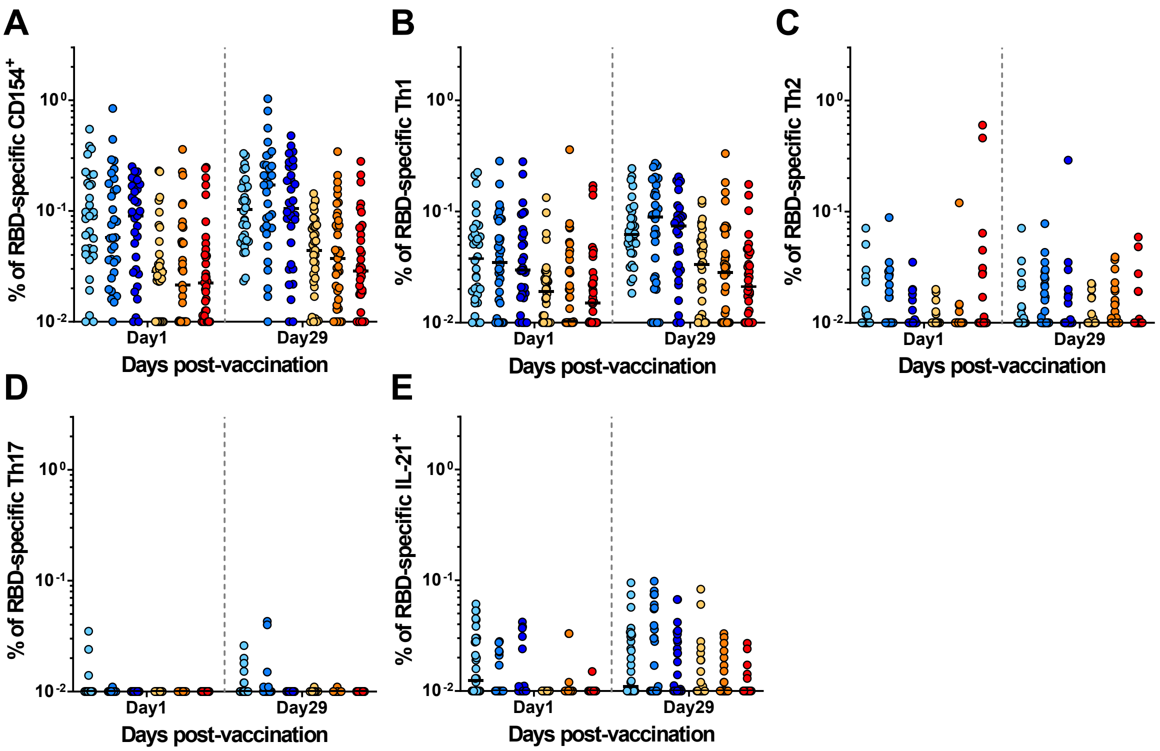

Flow cytometric analysis was performed to measure gamma RBD-specific T cells responses. Responses to 3 µg (Non-elderly participants: n = 32, Elderly participants: n = 34) or 7.5 µg (Non-elderly participants: n = 32, Elderly participants: n = 37) of VLPCOV-02 , or 30 µg of Comirnaty RTU (Non-elderly participants: n = 31, Elderly participants: n = 35) are shown as absolute numbers of responses on baseline (day 1) and week 4 (day 29). Panel A shows the activated $CD4^+$ T-cells, which was characterized by the expression of $CD154^+$. Panel B shows the response in $CD4^+$ Th1 cells, which was characterized by the expression of IL-2, TNF, and/or IFN-γ. Panel C shows the response in $CD4^+$ Th2 cells, which was characterized by expression of IL-4 and/or IL-13. Panel D shows the response in $CD4^+$ Th17 cells, characterized by the expression of IL-17. The Panel E shows the $CD4^+$ $IL\text{-}21^+$ cells, which was characterized by expression of IL-21. The horizontal bars indicate median values. Values below 0.01% were plotted at 0.01% as the minimum displayed value. However, the limit of detection (LOD) has not been determined in this analysis. *P* values (two-sided) were calculated using the Mann–Whitney *U*-test.

Figure S3. CD4⁺ T-cell responses (Fold change) against Spike XBB.1.5, related to Results

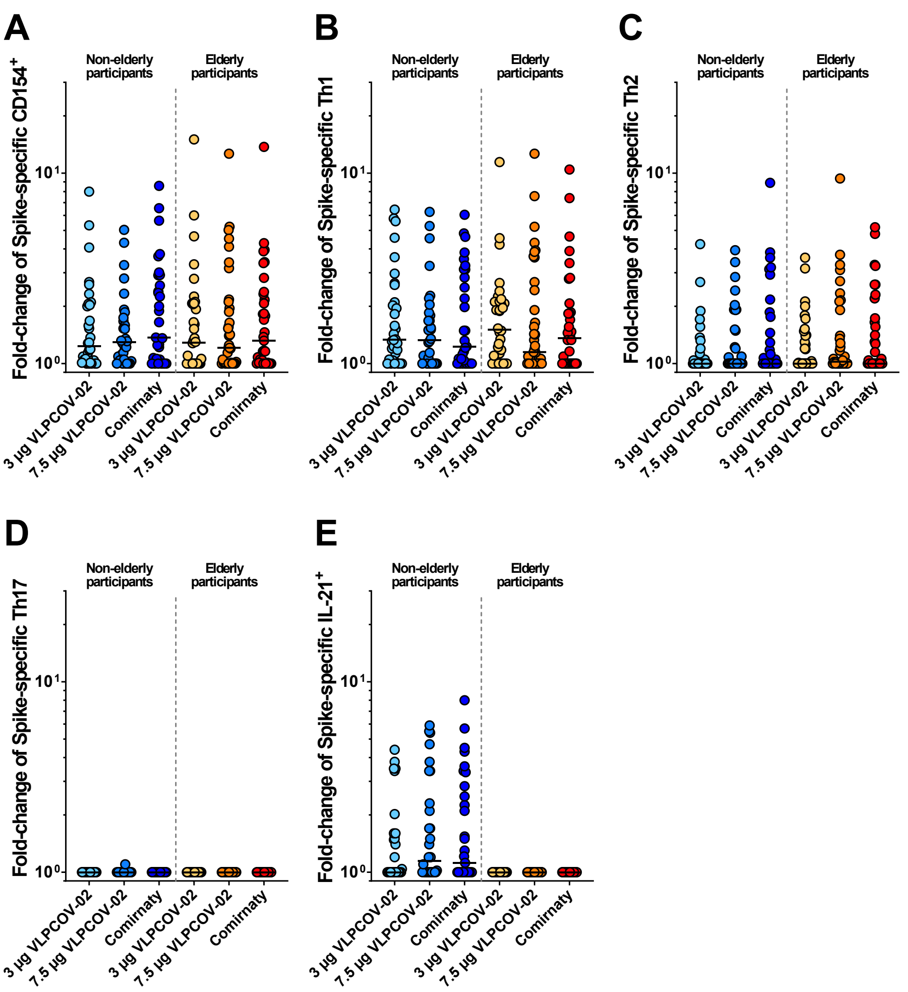

Flow cytometric analysis was performed to analyze XBB.1.5 Spike-specific T-cell responses. Responses to 3 µg (Non-elderly participants: n = 32, Elderly participants: n = 34) or 7.5 µg (Non-elderly participants: n = 32, Elderly participants: n = 37) of VLPCOV-02, or 30 µg of Comirnaty RTU (Non-elderly participants: n = 31, Elderly participants: n = 35) are shown as fold-change from baseline (day 1) to week 4 (day 29) for each cohort. Panel A shows the activated CD4⁺ T-cells, which was characterized by the expression of CD154⁺. Panel B shows the response in CD4⁺ Th1 cells, which was characterized by the expression of IL-2, TNF, and/or IFN-γ. Panel C shows the response in CD4⁺ Th2 cells, which was characterized by expression of IL-4 and/or IL-13. Panel D shows the response in CD4⁺ Th17 cells, characterized by the expression of IL-17. Panel E shows the CD4⁺ IL-21⁺ cells, which was characterized by expression of IL-21. The horizontal bars indicate median values. *P* values (two-sided) were calculated using the Mann–Whitney *U*-test.

Figure S4. CD4+ T-cell responses (Frequency) against Spike XBB.1.5, related to Results

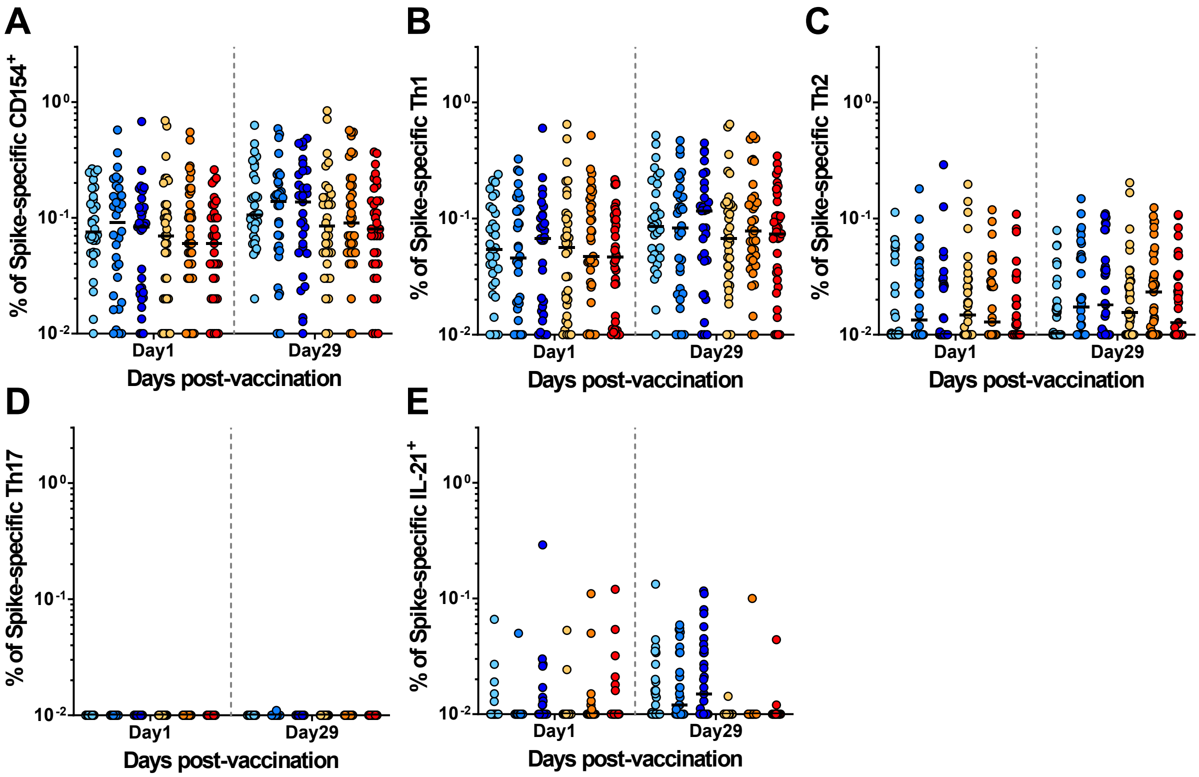

Flow cytometric analysis was performed to measure XBB.1.5 spike-specific T cells responses. Responses to 3 µg (Non-elderly participants: n = 32, Elderly participants: n = 34) or 7.5 µg (Non-elderly participants: n = 32, Elderly participants: n = 37) of VLPCOV-02, or 30 µg of Comirnaty RTU (Non-elderly participants: n = 31, Elderly participants: n = 35) are shown as absolute numbers of responses on baseline (day 1) and week 4 (day 29). Panel A shows the activated CD4+ T-cells, which was characterized by the expression of CD154+. Panel B shows the response in CD4+ Th1 cells, which was characterized by the expression of IL-2, TNF, and/or IFN-γ. Panel C shows the response in CD4+ Th2 cells, which was characterized by expression of IL-4 and/or IL-13. Panel D shows the response in CD4+ Th17 cells, characterized by the expression of IL-17. Panel E shows the CD4+ IL-21+ cells, which was characterized by expression of IL-21. The horizontal bars indicate median values. Values below 0.01% were plotted at 0.01% as the minimum displayed value. However, the limit of detection (LOD) has not been determined in this analysis. *P* values (two-sided) were calculated using the Mann–Whitney *U*-test.

Figure S5. Frequency of CD8⁺ T-cell responses against RBD gamma, related to Figure 6

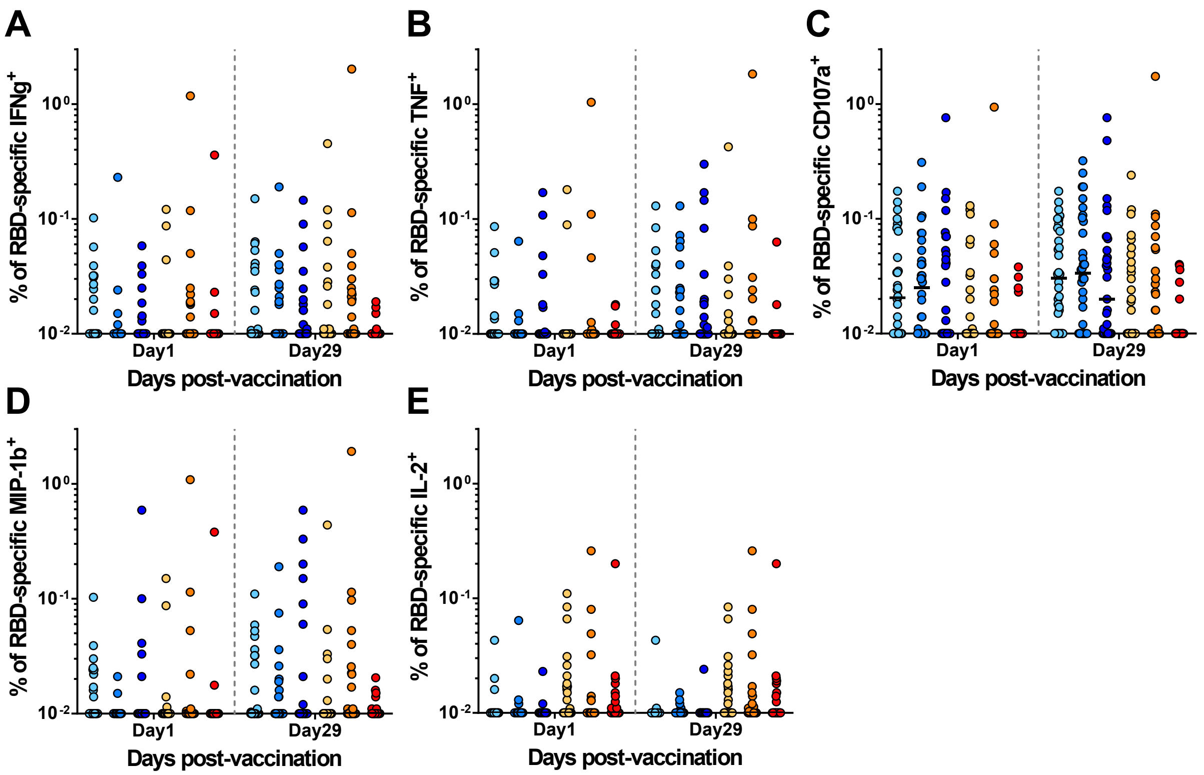

Flow cytometric analysis was performed to measure gamma RBD-specific T cells responses. Responses to 3 μg (Non-elderly participants: n = 32, Elderly participants: n = 34) or 7.5 μg (Non-elderly participants: n = 32, Elderly participants: n = 37) of VLPCOV-02, or 30 μg of Comirnaty RTU (Non-elderly participants: n = 31, Elderly participants: n = 35) are shown as absolute numbers of responses on baseline (day 1) and week 4 (day 29). Panels show the $CD8^+$ cells expressing IFN-γ (A), TNF (B), CD107a (C), MIP-1β (D), or IL-2 (E). The horizontal bars indicate median values. Values below 0.01% were plotted at 0.01% as the minimum displayed value. However, the limit of detection (LOD) has not been determined in this analysis. *P* values (two-sided) were calculated using the Mann–Whitney *U*-test.

Figure S6. CD8^{+} T-cell responses (Fold change) against Spike XBB.1.5, related to Results

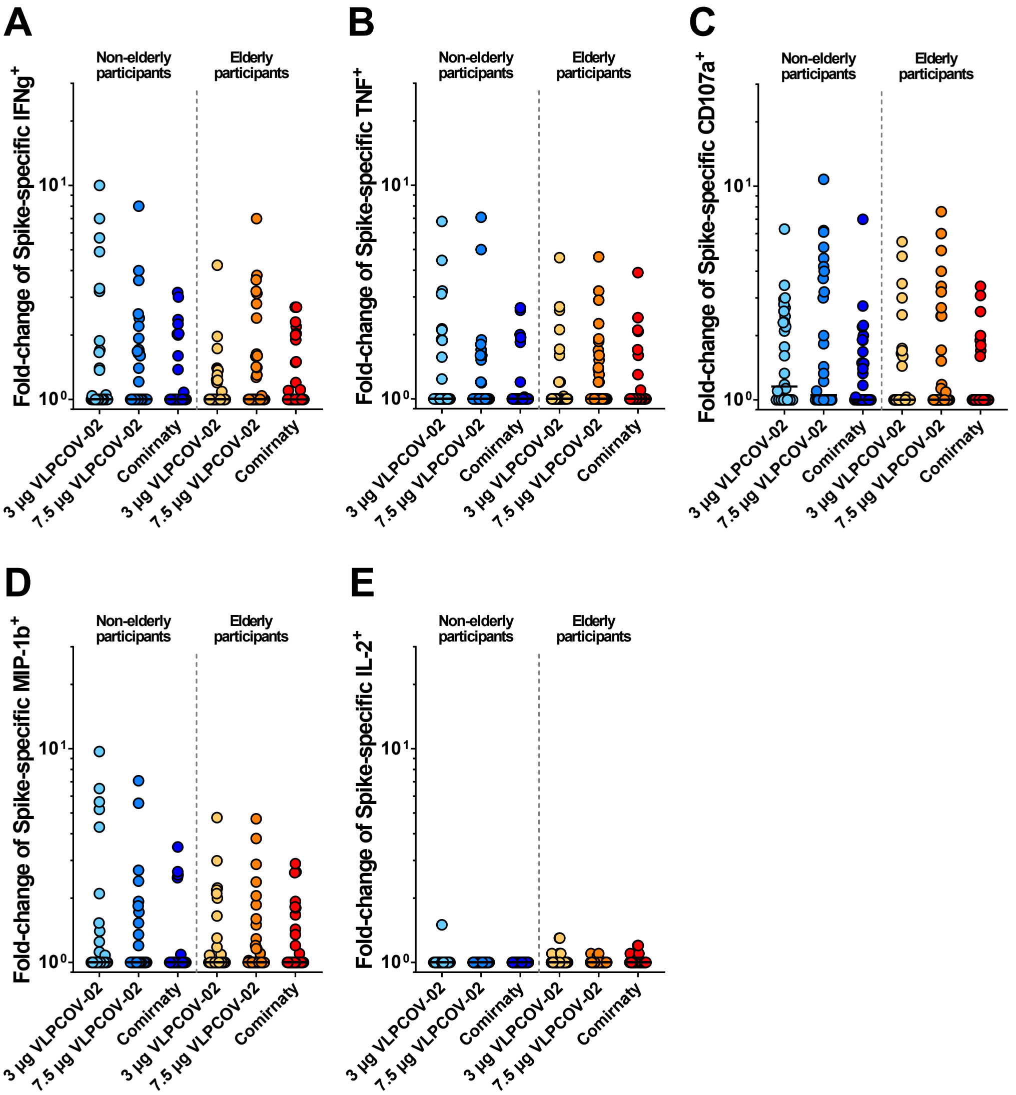

Flow cytometric analysis was performed to analyze XBB.1.5 Spike-specific T cells. Responses to 3 µg (Non-elderly participants: n = 32, Elderly participants: n = 34) or 7.5 µg (Non-elderly participants: n = 32, Elderly participants: n = 37) of VLPCOV-02, or 30 µg of Comirnaty RTU (Non-elderly participants: n = 31, Elderly participants: n = 35) are shown as fold-change from baseline (day 1) to week 4 (day 29). Panels show the CD8^{+} cells expressing IFN-γ (A), TNF (B), CD107a (C), MIP-1β (D), or IL-2 (E). The horizontal bars indicate median values. *P* values (two-sided) were calculated using the Mann–Whitney *U*-test.

Figure S7. CD8+ T-cell responses (Frequency) against Spike XBB.1.5, related to Results

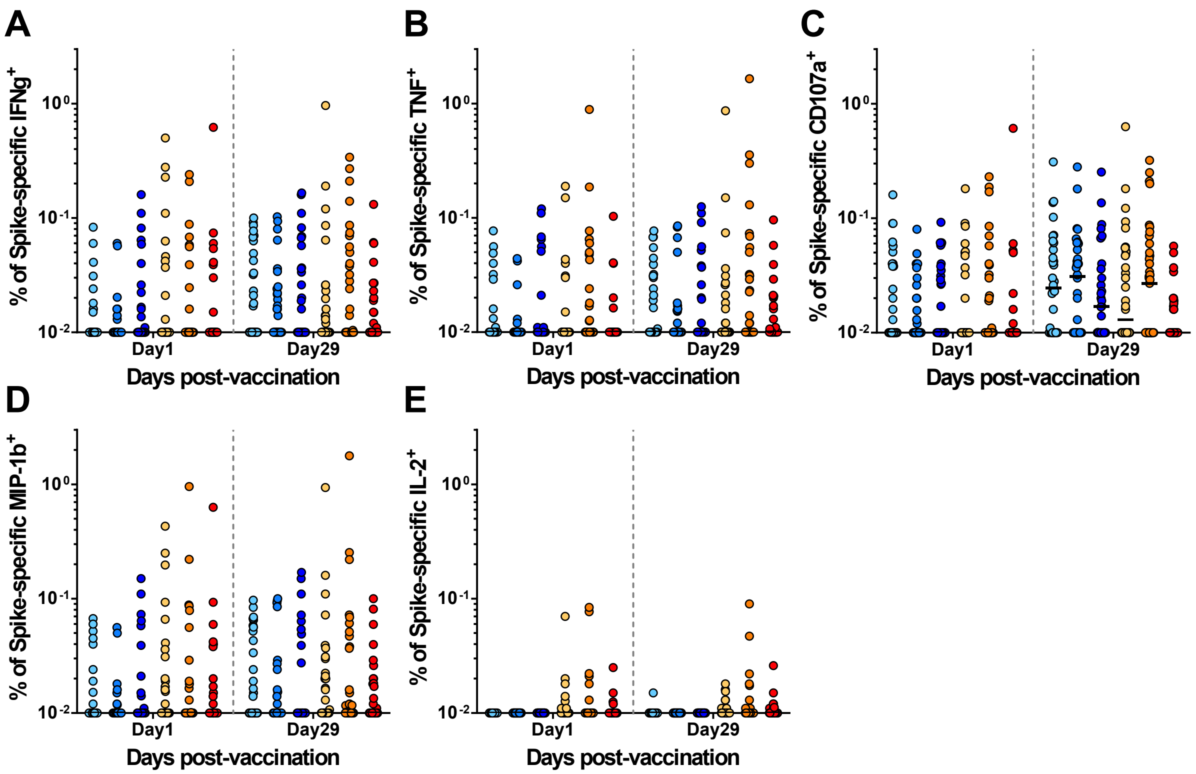

Flow cytometric analysis was performed to measure XBB.1.5 spike-specific T cells responses. Responses to 3 μg (Non-elderly participants: n = 32, Elderly participants: n = 34) or 7.5 μg (Non-elderly participants: n = 32, Elderly participants: n = 37) of VLPCOV-02, or 30 μg of Comirnaty RTU (Non-elderly participants: n = 31, Elderly participants: n = 35) are shown as absolute numbers of responses on baseline (day 1) and week 4 (day 29). Panels show the CD8+ cells expressing IFN-γ (A), TNF (B), CD107a (C), MIP-1β (D), or IL-2 (E). The horizontal bars indicate median values. Values below 0.01% were plotted at 0.01% as the minimum displayed value. However, the limit of detection (LOD) has not been determined in this analysis. *P* values (two-sided) were calculated using the Mann–Whitney *U*-test.

Figure S8. Representative gating strategy for T cell analysis, related to STAR Methods

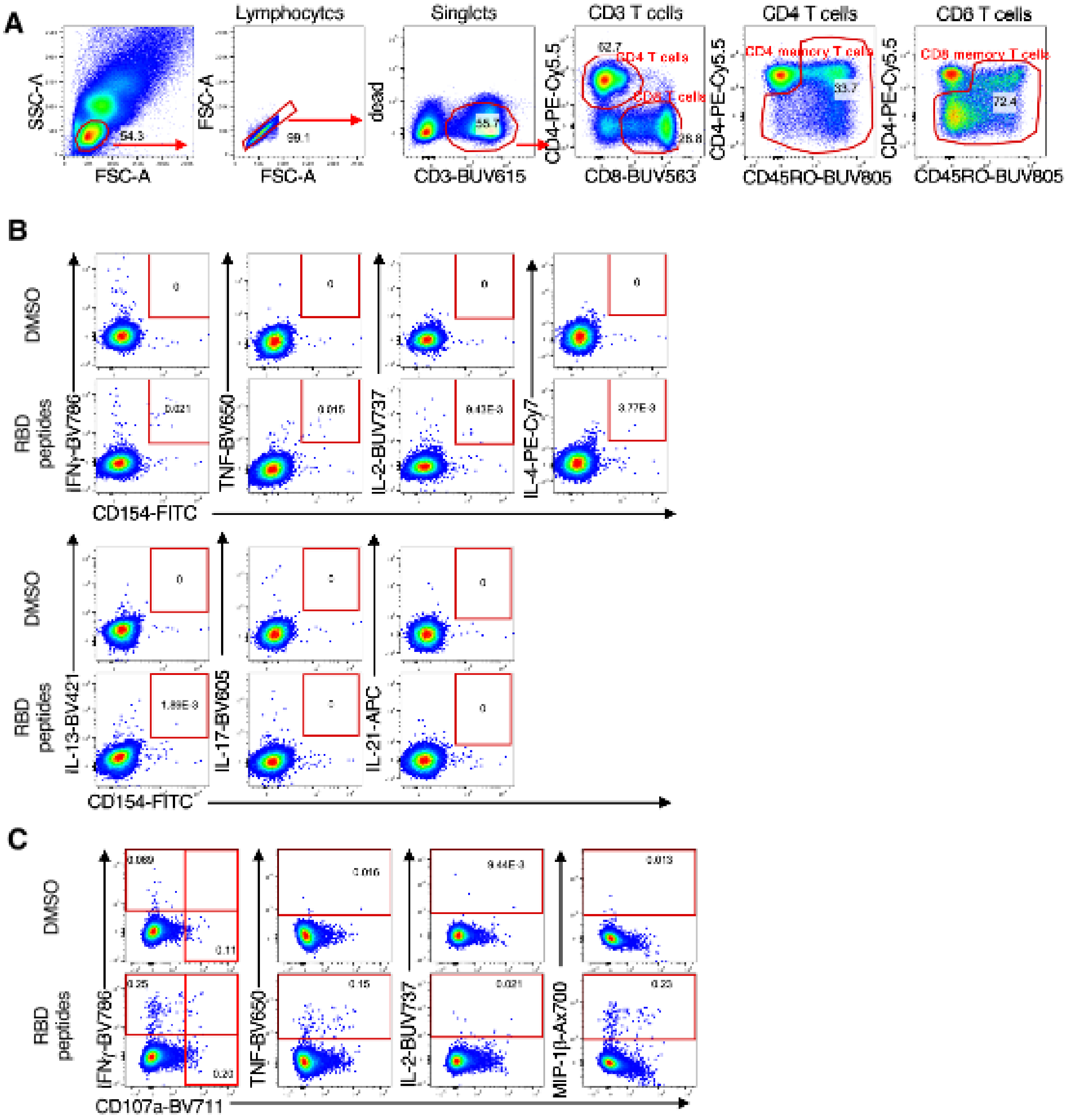

A) The top 6 panels show the strategy for separation of the peripheral blood mononuclear cells (PBMCs) into CD4⁺ and CD8⁺ T-cells. Subsequently, CD4⁺ and CD8⁺ T-cells were further divided into memory phenotypes based on the expression of CD27 and CD45RO. The representative flow cytometry gating strategies were generated using human samples from the 7.5 μg VLPCOV-02 vaccination group.

B) The panels show the gating strategy for RBD-specific cells expressing various phenotypic markers (CD154, IFN-γ, TNF, IL-2, IL4, IL-13, IL-17 and IL-21) in $CD4^+$ total memory cells. The background of frequencies of cytokine production (measured in DMSO control) were subtracted. The representative flow cytometry gating strategies were generated using human samples from the 7.5 µg VLPCOV-02 vaccination group.

C) The panels show the gating strategy for RBD-specific cells expressing various phenotypic markers (IFN-γ, TNF, CD107a, MIP-1β and IL-2) in $CD8^+$ total memory cells. The background of frequencies of cytokine production (measured in DMSO control) were subtracted. The representative flow cytometry gating strategies were generated using human samples from the 7.5 µg VLPCOV-02 vaccination group.
